# The Effects of Qinghao-Kushen and Its Active Compounds on the Biological Characteristics of Liver Cancer Cells

**DOI:** 10.1155/2022/8763510

**Published:** 2022-06-10

**Authors:** Jingmin Ji, Zhiqin Zhang, Qing Peng, Liyuan Hao, Yinglin Guo, Yu Xue, Yiwei Liu, Caige Li, Xinli Shi

**Affiliations:** Department of Pathobiology and Immunology, Hebei University of Chinese Medicine, Shijiazhuang 050200, China

## Abstract

**Background and Aims:**

*Artemisia annua* (Qinghao) and *Sophora flavescens* (Kushen) are traditional Chinese medicines (TCMs). They are widely used in disease therapy, including hepatocellular carcinoma (HCC). However, their key compounds and targets for HCC treatment are unclear. This article mainly analyzed the vital active compounds and the mechanism of Qinghao-Kushen acting on HCC.

**Methods:**

First, we chose a traditional Chinese medicine, which has an excellent clinical effect on HCC by network meta-analysis. Then, we composed the Qinghao-Kushen herb pair and prepared the medicated serum. The active compounds of Qinghao-Kushen were verified by the LC-MS method. Next, we detected key targets from PubChem, SymMap, SwissTargetPrediction, DisGeNET, and GeneCards databases. Subsequently, the mechanism of Qinghao-Kushen was predicted by network pharmacology strategy and primarily examined in HuH-7 cells, HepG2 cells, and HepG2215 cells.

**Results:**

The effect of the Qinghao-Kushen combination was significantly better than that of single Qinghao or single Kushen in HepG2 and HuH-7 cells. Qinghao-Kushen increased the expression of activated caspase-3 protein than Qinghao or Kushen alone in HepG2 and HepG2215 cells. Network analyses and the LC-MS method revealed that the pivotal compounds of Qinghao-Kushen were matrine and scopoletin. GSK-3*β* was one of the critical molecules related to Qinghao-Kushen. We confirmed that Qinghao-Kushen and matrine-scopoletin decreased the expression of GSK-3*β* in HepG2 cells while increased GSK-3*β* expression in HepG2215 cells.

**Conclusions:**

This work not only illustrated that the practical components of Qinghao-Kushen on HCC were matrine and scopoletin but shed light on the inhibitory of Qinghao-Kushen and matrine-scopoletin on liver cancer cells. Moreover, Qinghao-Kushen and matrine-scopoletin had a synergistic effect over the drug alone in HuH-7, HepG2, or HepG2215 cells. GSK-3*β* may be a potential target for HCC therapy.

## 1. Introduction

Liver cancer is the second lethal tumor in China. Hepatocellular carcinoma (HCC) is a malignant tumor of the liver, representing 85–90% of primary liver cancer [1]. Chronic hepatitis B virus (HBV) infection is the leading risk factor for HCC. Early stage HCC is treated by surgical resection, radiotherapy and chemotherapy techniques, and liver transplantation. But most patients with HCC are in the advanced stage [2]. Oxaliplatin-based chemotherapy is used to treat patients with advanced HCC [3]. However, the chemoresistance of oxaliplatin is the main factor restricting its therapeutic effect [4, 5]. As an important alternative and complementary drug, Chinese medicine improves the efficacy of systemic chemotherapy effectively and reduces its adverse reactions [6, 7], and it has been used around the world [8, 9].

*Artemisia annua L* (Qinghao) is the dry aboveground plant of the *Artemisia annua* (family Compositae) [10], with a common name sweet wormwood herb [11, 12]. The plant first appears in the “Shennong herbal Scripture” (Shen nong ben cao jing) [13], Then, Tu et al. found that *Artemisia annua* was used to treat plasmodia infections [14]. Qinghao and its derivatives are not limited to treat malaria but also have a role in treating cancer [11, 15, 16]. Our previous study showed that dihydroartemisinin (DHA) effectively reduced cell proliferation in HepG2215 cells [17]. Most of Qinghao's drugs are combination therapies rather than single-drug therapy in the clinical [18]. *Sophora flavescens* (Kushen) is the dried root of the *Sophora flavescens* plant [10], with a common name of the root of light-yellow sophora [19]. It is in the genus *Sophora* (family *Fabaceae Lindl*) [20]. The function of *Sophora flavescens* is recorded in the “Shennong herbal Scripture” (Shen nong ben cao jing) [13]. Kushen contains alkaloids and flavonoids. Matrine is the primary alkaloid component found in the roots of Kushen [21]. Kushen has anti-liver cancer and other pharmacological effects [22]. Furthermore, matrine promotes the apoptotic rate in HepG2 cells [23]. GSK-3*β* (glycogen synthase kinase 3 beta) is a protein-coding gene. It is a ubiquitously expressed serine-threonine kinase. GSK-3*β* has many biologic functions, including energy metabolism, inflammation, and mitochondrial dysfunction [24]. In some cases, GSK-3*β* not only has tumor suppressor function, but it also has tumor promoter function [25].

In this study, we combined Qinghao-Kushen. Then, identified the active compounds of Qinghao-Kushen by the LC-MS method. Furthermore, we confirmed that Qinghao-Kushen inhibited proliferation and viability in HepG2, HuH-7, and HepG2215 cells. Our work provided an effective strategy for revealing the mechanism of Qinghao-Kushen at molecular, cellular, and biologic levels.

## 2. Materials and Methods

### 2.1. Search Strategy and Data Extraction

Our study included only randomized controlled trials of HCC. We selected Chinese medicine combined with FOLFOX (oxaliplatin-based chemotherapy drugs) therapy for the experiment group. FOLFOX therapy was chosen for the control group. We used a combination of free words and Medical Subject Heading terms to search. The terms were searched in the PubMed, Cochrane, Wan-Fang Database, Chinese National Knowledge Infrastructure (CNKI), and China Biology Medicine Disc (CBM) from their start to May 30, 2020, and included the following: liver cancer, hepatocellular carcinoma, traditional Chinese medicine, Chinese herbal, and FOLFOX.

Two authors (JJ and ZZ) screened the preliminary literature following the inclusion independently. After excluding irrelevant literature, the full text was browsed to determine the final included literature. The same authors collected the data, including author, sample size, interventions, and outcomes. We used the risk of bias assessment tool from the Cochrane Collaboration to check the risk of bias.

### 2.2. Preparation of Drug-Containing Serum, Matrine, and Scopoletin

We purchased 32 male Sprague Dawley rats (6–8 weeks, 180–200 g) from Beijing Vital River Laboratory Animal Technology Co., Ltd (laboratory animal production license number: SCXK (Jing) 2016-0006). Ethical approval for the study was granted by the Ethics Committee of Hebei University of Chinese Medicine (Yxll2021040). The rats were kept in a clean animal room with 12-h dark and 12-h light. Rats were left freely to eat and drink. SD rats were randomly divided into four groups. Qinghao formula granules (20080261) were purchased from the National Medical Hall of Hebei University of traditional Chinese medicine. Qinghao formula granules were administered orally to Qinghao group rats (medicinal slices 2.7 g/kg). The size per gram of Qinghao formula granules was equal to 14.3 per gram of medicinal tablets. Similarly, we purchased Kushen formula granules (20082661) from the National Medical Hall of Hebei University of Traditional Chinese Medicine. Kushen formula granules were administered orally to Kushen group rats (medicinal slices 1.44 g/kg). The size per gram of Kushen formula granules was equal to 12 per gram of medicinal tablets. Qinghao and Kushen were administered orally to Qinghao-Kushen group rats. The same dose of normal saline was administered orally to blank serum group rats. Twice a day for 4 days, the rats were anesthetized with pentobarbital (100 mg/kg) 1 hour after the last administration. Blood was collected from the abdominal aorta in a sterile manner, which was then centrifuged and bathed at 56°C and finally filtered, packed, and stored at −80°C for further use. Matrine and scopoletin were purchased from MedChemExpress Co., Ltd (Monmouth Junction, NJ, USA) and dissolved these drugs with dimethyl sulfoxide (DMSO) (Sigma, Aldrich; Merck KGaA).

### 2.3. Serum LC-MS Metabolic Experiment Analysis

We prepared drug-containing serums. Then the serums were sent to the Meiji Biotechnology for total compound testing (Shanghai Meiji Biotechnology Co., Ltd., Shanghai, China). 100 *μ*L of drug-containing serums was measured from different groups (Qinghao medicated serum, Kushen medicated serum, herb pair of Qinghao-Kushen medicated serum, and normal saline serum). Raw data were imported into the metabonomics processing software Progenesisqi (Waterscorporation, Milford, USA) for base analysis to identify the characteristic peak search database and analyze the metabolic numbers. The mass error of MS was set to less than 10 PPM, and the metabolites were identified according to the matching score of secondary mass spectrometry. The central database is https://www.hmdb.ca/ and https://metlin.scripps.edu/.

### 2.4. Target Prediction of Active Compounds and Collection of HCC Therapeutic Genes

We predicted the targets of the compounds from four databases. PubChem is the world's largest free chemical information site, and the targets were reported in the literature (https://pubchem.ncbi.nlm.nih.gov/). SymMap combines TCM with modern medicine through internal molecular mechanisms and external symptom mapping. Besides, it provides external links to TCMSP and TCMID (https://www.symmap.org/). FAFDrugs4 searches the ADME information of the compounds. A drug-like soft criterion was used to screen the ADME information of the compounds. The compounds that passed the standard were finally selected (https://fafdrugs4.mti.univ-paris-diderot.fr). In SwissTargetPrediction, target prediction was based on structural similarity (https://swisstargetprediction.ch/).

We collected HCC-related targets from two sources. GeneCards provides human genes (https://www.genecards.org/) [26]. DisGeNET database screens genes according to Disease Pleiotropy Index (DPI) and Disease Specificity Index (DSI) (https://www.disgenet.org/).

### 2.5. Cell Culture

Human hepatoma HepG2 and HepG2215 cell lines and normal human liver L02 cells were purchased from American Type Culture Collection (ATCC; Manassas, VA). HCC HuH-7 cells were purchased from Procell Life Science & Technology Co., Ltd (Procell; Wuhan, China). HepG2215 cells are HBV-transfected HepG2 cells that can replicate HBV [27]. The cells were cultured in Dulbecco's Modified Eagle Medium (DMEM) (Gibco, USA) containing 10% fetal bovine serum (Gibco, USA). All cells were cultured in a carbon dioxide incubator having a humid atmosphere of 37°C and 5% CO_2_.

### 2.6. CCK8 Assay

We used the CCK8 kit (Share-Bio, China) to measure the viability of HepG2, HuH-7, and HepG2215 cells. The cells were placed in 96-well plates with a volume of 100 *μ*L in each well (1 × 10^4^ cells/well). After the cells adhered to the wall, the cells were treated with different culture mediums for 12 h, 24 h, and 36 h. Three replicate wells were set in each concentration. 10 *μ*L of CCK8 solution was added to each well and then incubated in a carbon dioxide incubator for 2 hours. Finally, the OD value was measured at 450 nm by the Multiskan Spectrum Microplate Reader (Thermo Scientific, USA). IC_50_ was calculated according to OD value.

### 2.7. Cytotoxicity of Medicated Serum

A CCK8 kit was used to detect the cytotoxicity of different medicated serum on L02 cells. L02 cells were inoculated in a 96-well plate as described above. After the cells were attached, L02 cells were treated with 24-hour IC_50_ concentration. After 24 hours of culture, the OD value was measured at 450 nm to calculate cell viability.

### 2.8. Cloning Formation Assay

HepG2 cell were inoculated into 6-well plates. Each well contained 2 mL of the complete medium. After adhering to the wall, the cells were treated with different drugs and cisplatin (5.0 *μ*M DDP) for 24 h. After 24 hours of various interventions, the cells were resuspended and counted. A total of 300 cells were inoculated into 6-well plates. Three replicate wells were set for each intervention. Finally, sufficient complete medium was added to the culture cells for 14 days after which cell cloning was observed.

### 2.9. Cell Migration Assay

HuH-7, HepG2, and HepG2215 cells were seeded into 12-well plates. After adhering to the wall, the cells were scratched with 100 *μ*L pipette tips. The exfoliated cells were washed with PBS. Then, three cells were treated with different medicated serum and DDP. Cell scratch healing was observed under a microscope at 0 h and 24 h, respectively. Cell migratory ability (%) = (0-h scratch area – 24-h scratch area)/0-h scratch area × 100%. The experiment was repeated three times for each group.

### 2.10. Transwell Invasion Assays

For invasion assays, transwell chambers were covered with Matrigel (BD Falcon, NY, USA). We diluted it with serum-free DMEM (Matrigel: DMEM = 1 : 8). HuH-7 cells were treated with different medicated serum and DDP for 24 h. Then, the cells were cultured in the upper chamber of the transwell chambers (8-mm pore size, Corning, USA), which have 100 *μ*L Matrigel. DMEM containing 10% FBS was added to the bottom room. After culturing at 37°C with 5% CO_2_ for 24 h, the cells were cleaned twice with PBS, fixed with methanol, and stained with Giemsa. Finally, we used a microscope to observe the cells passing through the pore.

### 2.11. Western Blot

We extracted cell protein after different treatments (treated as described above) using Cell Protein Extraction Kit. (EpiZyme, China). A lysis buffer containing protease inhibitors (1 : 100) was used to lyse the cells. The supernatant was then collected after high-speed centrifugation (14000∼16000 rpm). Finally, proteins were mixed with loading buffer and denatured at 100°C for 5 min. ExpressPlus-PAGE (GenScript China) separated an equal amount of proteins and transferred them to the PVDF membrane. The primary antibodies were rabbit anti-GSK-3*β* monoclonal antibody (#12456, CST, diluted at 1 : 1000), rabbit anti-activated caspase-3 monoclonal antibody (CY5501, Abways, 1 : 1000), rabbit anti-GAPDH polyclonal antibody (ab0037, 1 : 5000), and anti-*β*-tubulin monoclonal antibody (#2146, CST, 1 : 5000). The primary antibodies were incubated overnight in a 4°C refrigerator. Then, they were incubated with the secondary antibody for 2 hours. The secondary antibody was goat anti-rabbit IgG-HRP (ZB-2301, ZSGB-BIO, 1 : 5000). The ECL detection system (Vilber, Fusion FX5, Spectra, France) was used to detect the bands. ImageJ software was used to measure the protein expression.

### 2.12. Molecular Docking

The essential targets predicted by the above experimental methods were selected for molecular docking with the main active compounds in Qinghao-Kushen. The molecular structure of the Qinghao-Kushen active ingredient was downloaded from the PubChem database (https://pubchem.ncbi.nlm.nih.gov/). Chem3D was used for format conversion and energy minimization. The target protein structure was obtained from the RCSB database (https://www.rcsb.org/). The Maestro11.9 platform was used to process protein structures. Then, molecular docking was done by the Glide module in Schrodinger Maestro software. The output of molecular docking structure was visualized using PyMOL.

### 2.13. Statistical Analysis

All data were presented as mean ± SD, which was collected after three independent experiments. Comparison between the two groups was performed by *t*-test, while one-way analysis of variance was used for comparison between two or more sample groups. The *P* < 0.05 was considered statistically significant.

## 3. Results

### 3.1. Qinghao-Kushen Was a Potential Effective Herb Pair for the Treatment of HCC by Network Meta-Analysis

**Figure 1:**
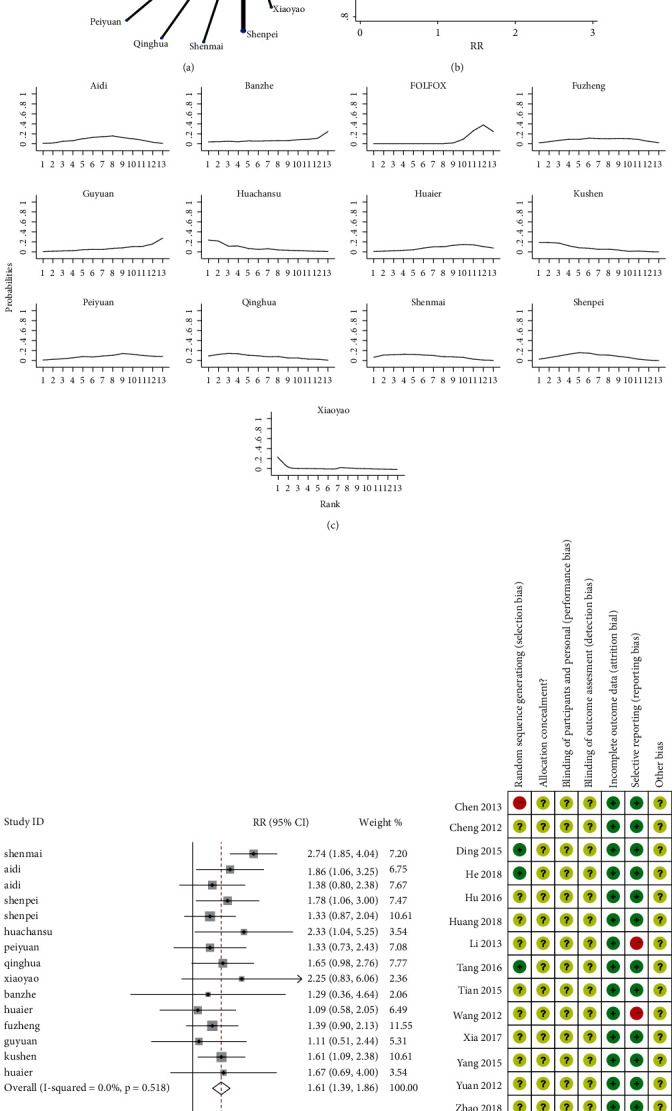
Qinghao-Kushen was a potential effective herb pair for the treatment of HCC by network meta-analysis. (a) Network analyses of total clinical efficiency. A line connected interventions that were directly compared. The thickness of the lines represents the number of studies. The size of the nodes corresponds to the total sample size of the interventions. (b) Funnel plot of comparison of overall efficacy. (c) Ranking for overall efficacy comparison of different interventions. Ranking indicates the probability of being the best treatment. (d) Forest plot of traditional meta-analysis. RR ＞ 1, the higher the RR (rate ratio) value, the better the efficacy of TCM treatment compared with the control group. (e) Risk of bias review authors' judgments about each risk of bias item. The red circle represents a high risk of bias, the yellow circle represents an unclear risk of bias, and the green circle represents a low risk of bias.

To assess the effects of different Chinese medicines on HCC, we did a multiple treatment meta-analysis. The data showed that there were 1221 references in the initial examination. Finally, 15 references that met the standards were included (Table S1). These contained 12 interventions with 1090 patients, namely Kushen injection, Shenmai injection, Aidi injection, Shenpei soup, Cinobufacini injection (Huachansu), Peiyuan anticancer soup, Qinghua anticancer soup, Xiaoyao soup, Compound Cantharis injection (Banzhe), Huaier granule, Fuzheng Jiedu decoction, and Guyuan Jiedu decoction (Table S2). The result showed that Kushen was one of the candidates to form herb pair with Qinghao. To assess the quality of the literature included in the study, we did the risk of bias assessment (Figure 1(e)). The data showed that three studies were rated to a low risk of bias because of a random number. Two studies were judged a high risk of bias because of the wrong randomization method. A comparison-corrected funnel plot (Figure 1(b)) was drawn, which used the overall effective rate as the outcome index. The funnel plot distribution was symmetrical, indicating that the possibility of a small sample effect was slight. These results showed that the literature of Kushen was reliable.

To analyze more effective traditional Chinese medicine, we did a network analysis of the total clinical efficiency (Figure 1(a)). The link between the two nodes indicated direct comparison evidence between the two interventions. The data showed that the efficacy of different Chinese medicines was compared with that of common positive control drugs. Therefore, we made a pairwise comparison of the effects from 12 traditional Chinese medicines for HCC treatment (Table 1). The results of network meta-analysis using Bayesian theory showed that Cinobufacini (Huachansu) injection (3.50, (1.11, 11.02)), Kushen injection (3.22, (1.27, 8.19)), Qinghua anticancer soup (2.54, (1.00, 6.45)), Shenmai injection (2.48, (1.06, 5.80)), and Shenpei soup (2.27, (1.20, 4.30)) were significantly more efficacious than the FOLFOX (*P* < 0.05). These outcomes were consistent with the findings of the simple pairwise meta-analyses (Figure 1(d)). Together, these results supported that the anti-HCC effect of Kushen combined with FOLFOX was better than that of FOLFOX alone.

Finally, the probabilities of different treatments were sorted to the 13 possible locations (Figure 1(c)). Meanwhile, Table S3 supplied the rank of SUCRA results for various interventions. Both results showed that Cinobufacini (Huachansu) injection (SUCRA = 0.775) and Kushen injection (SUCRA = 0.77) had the best efficacy in the treatment of HCC patients. In short, Kushen and Huachansu were the most effective for treating HCC, but the mechanism of Kushen was unknown. Therefore, we chose Kushen as the object. DHA was a crucial compound in *Artemisia annua* (Qinghao). Our previous research indicated that DHA suppressed cell proliferation in HepG2215 cells in a dose- and time-dependent manner [17]. We tried to find another clinical effective Chinese medicine for the treatment of HCC, which can combine with Qinghao. Previous studies showed that Hugan Badu Ointment with Qinghao and Kushen as the main components had definite clinical efficacy in treating chronic hepatitis B and improved liver function [28]. We explored the effectiveness of Qinghao and Kushen based on their effective improvement of liver function. In summary, Qinghao-Kushen was a potential practical herb pair for the treatment of HCC.

### 3.2. Thirty-One Metabolic Compounds in the Serum of Qinghao-Kushen Were Determined by LC-MS

To identify the active compounds of Qinghao-Kushen, we used the LC-MS method to detect compounds in the serum. (Figure S1). The data showed that the peak shape was good, and the distribution was relatively uniform under this detection condition.

Next, the LC-MS information was matched with the metabolic database to obtain the metabolites. The metabolites were selected according to fragmentation score (Metlin database) ≥30, theoretical fragmentation score (HMDB database) ≥30, and mass error (PPM) ＜10 (Table S4). There were 15 and 14 metabolized compounds in the Qinghao and Kushen, respectively. Moreover, these compounds were also in Qinghao-Kushen. Importantly, these compounds were not present in normal saline serum. It showed that these compounds might play an essential anticancer role in Qinghao-Kushen.

### 3.3. GSK-3*β* Was a Potential Target in Qinghao-Kushen by Network Analysis

**Figure 2:**
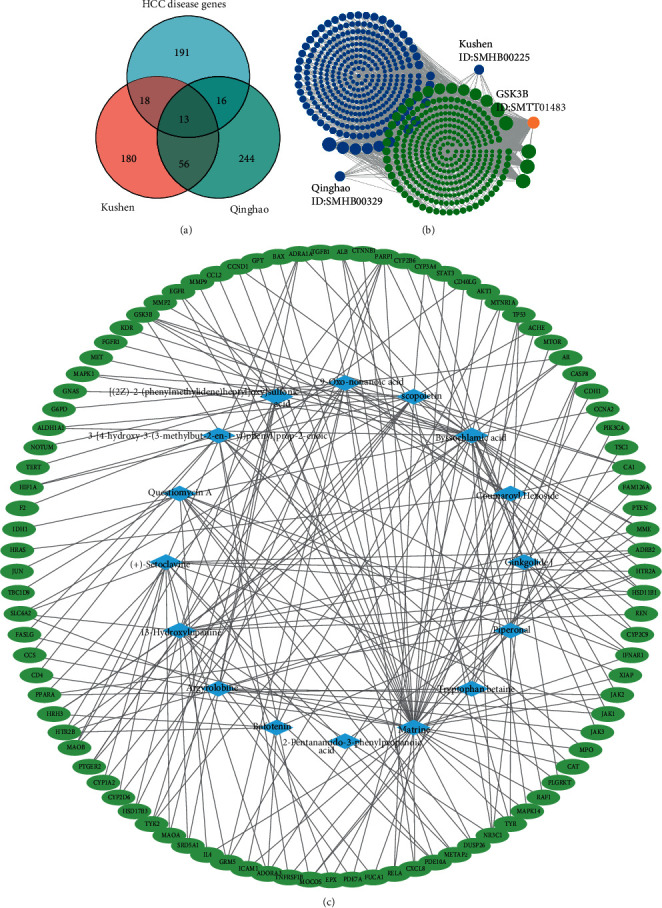
GSK-3*β* was a potential target in Qinghao-Kushen by network analysis. (a) The Venn diagram of targets of Qinghao, Kushen, and HCC. (b) GSK-3*β* interacting compounds and herbs in SymMap database. Orange represents GSK-3*β*, green represents compounds, and blue represents herbs. (c) Compound-target network, blue represents compounds, green represents targets.

To explore the critical targets of the active compounds and detect genes for HCC, we inputted the above compounds into PubChem, SymMap, and SwissTargetPrediction databases to retrieve the targets of Qinghao-Kushen. At the same time, we retrieved the DisGeNET database and GeneCards database to collect HCC disease targets. One hundred fifty-one targets for HCC were collected from the DisGeNET database. Meanwhile, 130 genes were selected from the GeneCards database according to＞the 8.8 scores. Finally, 238 HCC therapeutic targets were collected by removing duplicates (Figure 2(a)). In addition, we predicted 329 targets of Qinghao and 267 targets of Kushen from PubChem, SymMap, and SwissTargetPrediction. The Venn plot of the above gene (Figure 2(a)) showed that 47 targets were drug targets and therapeutic genes, considering them for subsequent research. Next, we built the compound-target network diagram (Figure 2(c)). The results showed that there were many compounds such as scopoletin, matrine, coumaroyl hexoside, byssochlamic acid, and {[(2Z)-2-(phenylmethylidene) heptyl]oxy}sulfonic acid that targeted GSK-3*β*. Last, we searched GSK-3*β* in the SymMap database. Interestingly, GSK-3*β* was the target of many compounds, some from Qinghao and others from Kushen (Figure 2(b)). In short, GSK-3*β* was a potential target of Qinghao-Kushen.

### 3.4. Qinghao-Kushen Had a Potential Biological Effect of Inhibiting Cell Proliferation by GO Enrichment Analysis

**Figure 3:**
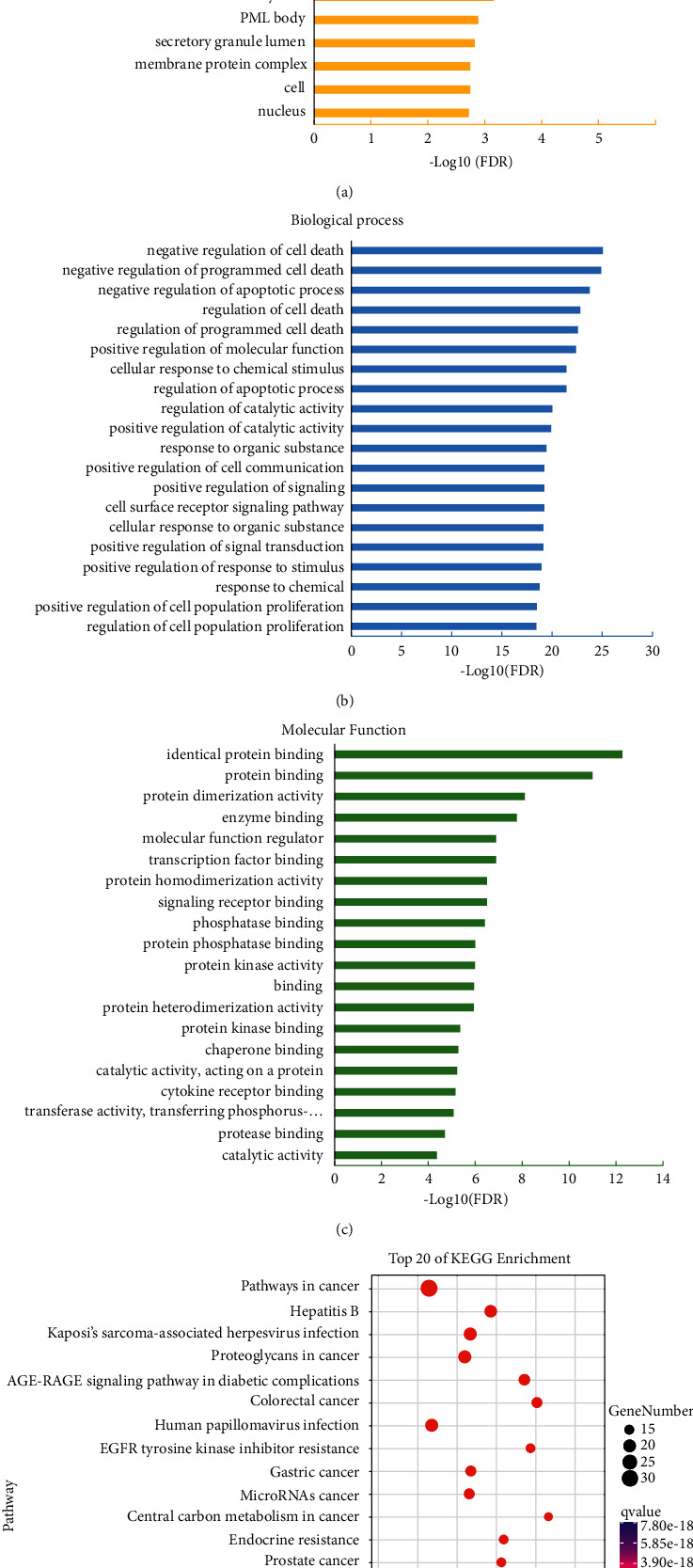
Qinghao-Kushen had a potential biological effect of inhibiting cell proliferation by GO enrichment analysis. (a) The top 20 GO items in cellular component. (b) The top 20 GO items in biological process. (c) The top 20 GO items in molecular function. (d) The top 20 GO items in KEGG pathway enrichment analyses.

To further explore the biological effects of the key targets on HCC, we entered 47 targets into the STRING database. The highest confidence (0.9) was set to evaluate the selected targets' biological process, cellular component, and molecular function. For enrichment analysis, the top 20 GO entries were chosen according to FDR< 0.01. The CC results (Figure 3(a)) mainly involved intracellular organelle, lumen, membrane raft, cytosol, intracellular extracellular region, and mitochondrion. The main terms of BP (Figure 3(b)) were cell population proliferation, cell death, and regulation of the apoptotic process. Most GO terms of MF (Figure 3(c)) were identical protein binding, enzyme binding, and molecular function regulators. The KEGG pathway annotation showed that 132 pathways were enriched by 47 targets (*P* < 0.001). The top 20 KEGG pathways (Figure 3(d)) with high counts included many cancer pathways, such as pathways in cancer and hepatocellular carcinoma pathway. In short, Qinghao-Kushen has a potential biological effect of inhibiting cell proliferation.

### 3.5. Matrine and Scopoletin Targeted GSK-3*β* by Analyzing Herb-Compound-Target-Pathway Networks

**Figure 4:**
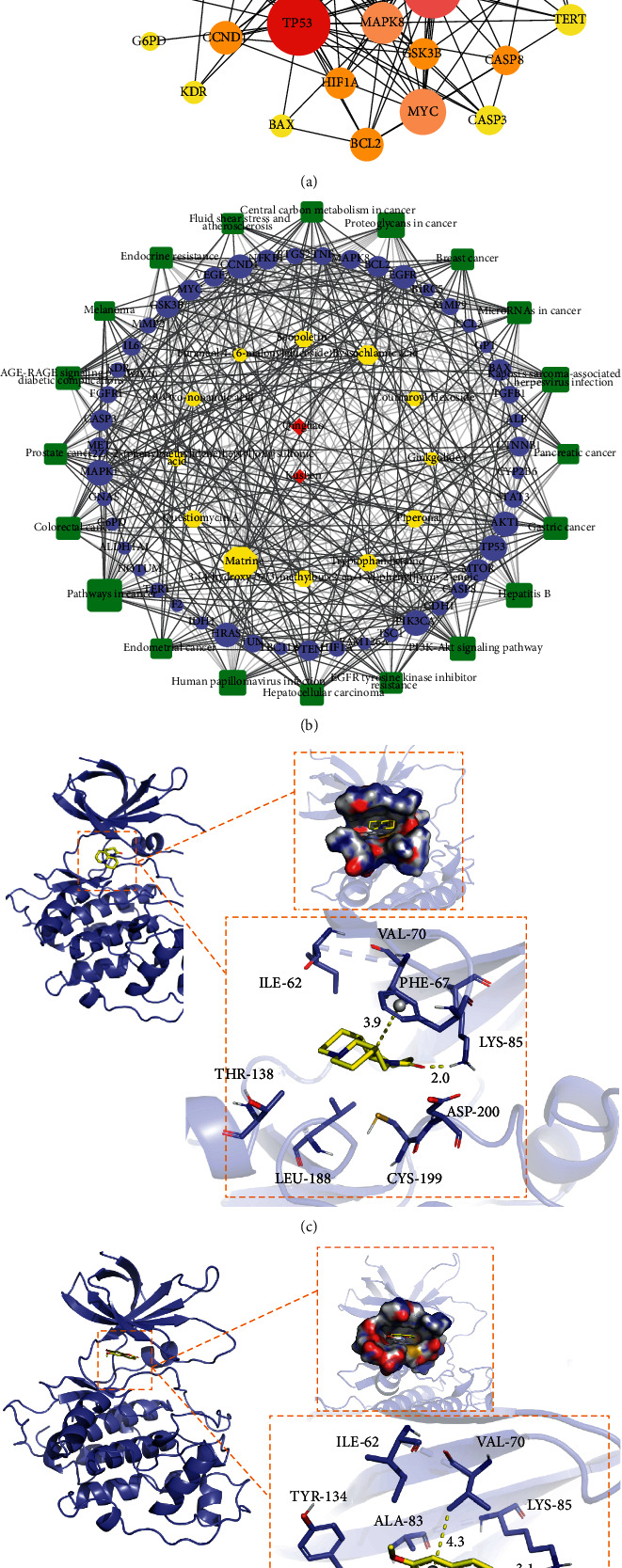
Matrine and scopoletin targeted GSK-3*β* by analyzing herb-compound-target-pathway networks. (a) Protein-protein interaction analysis. Larger nodes represent a higher degree of the targets, red represents the largest degree, and yellow represents a minor degree. (b) Herb-compound-target-pathway interaction network (H-C-T-P network). The node size is expressed in terms of degree. The larger the degree value, the larger the shape of the graph. (c) The binding mode of GSK-3*β* with matrine. (d) The binding mode of GSK-3*β* with scopoletin. The backbone of protein was rendered in tube and colored in blue. The compound is rendered in yellow. Yellow dash represents hydrogen bond distance.

To further elucidate the mechanism of Qinghao-Kushen, network topology analysis was carried out. The 47 key genes were input into the STRING database to obtain the protein-protein data. Then, these data were fed into Cytoscape 3.7.1 software for network topology analysis. The network (Figure 4(a)) included 45 nodes and 189 edges. The data showed that GSK-3*β*, STAT3, TP53, AKT1, MAPK1, and VEGFA were essential targets in the protein-protein network.

Meanwhile, we constructed an herb-compound-target-pathway interaction network (H-C-T-P network) (Figure 4(b)). It showed 81 nodes and 440 edges, and the H-C-T-P network contained two herbs, 12 compounds, 47 targets, and 20 pathways. The node size was expressed in degree, and the higher the degree value, the more critical the node was. The results indicated matrine and scopoletin were pivotal compounds in Qinghao-Kushen. Scopoletin (193.049 m/z, C10H8O4), which was identified in Qinghao-Kushen, was a bioactive compound of Qinghao. Scopoletin has many pharmacological effects, including antioxidative [29] and cytotoxicity towards cancer cells [30]. Furthermore, we detected matrine (249.195 m/z, C15H24N2O), a bioactive compound of Kushen, in Qinghao-Kushen (Table S4). In a clinical setting, matrine has long been the adjuvant therapy for liver cancer [31, 32]. Importantly, we found that GSK-3*β* was the target of matrine and scopoletin. Meanwhile, GSK-3*β* was related to many cancer pathways. In conclusion, matrine and scopoletin were essential active compounds targeting GSK-3*β* in Qinghao-Kushen.

We tested the precision of docking between matrine and scopoletin with GSK-3*β*. Matrine and scopoletin grafted with GSK-3*β* (PDB ID ：6Y9S) target protein and stably grafted into the active pocket of GSK-3*β* protein (binding energy less than -6 kcal/mol). The active pocket of matrine that combined with GSK-3*β* contained the following amino acids: LE-62, THR-138, LEU-188, CYS-199, ASP-200, LYS-85, PHE-67, and VAL-70 (length: <3.0 Å) (Figure 4(c)). Matrine was bound to GSK-3*β* by forming hydrogen bonds with LYS-85 (2.0 Å). In particular, PHE-67 can form *π*-*π* conjugated interaction with matrine and contribute to small molecules' stability. Similarly, scopoletin combined with GSK-3*β* contained the following amino acids: ILE-62, TYR-134, ALA-83, VAL-135, ASP-133, LEU-188, VAL-110, CYS-199, ASL-200, LEU-132, and LYS-83 (length: <3.0 Å) (Figure 4(d)). Scopoletin was bound to GSK-3*β* by forming hydrogen bonds with LYS-85, TYR-134, and ASP-133 (average length 2.43 Å). VAL-70 and LEU-188 can form *π*-*π* conjugated interaction with scopolamine. Overall, these results prove GSK-3*β* acted as Qinghao-Kushen targets.

### 3.6. Qinghao-Kushen Influenced the Expression of GSK-3*β* in Liver Cancer Cells

**Figure 5:**
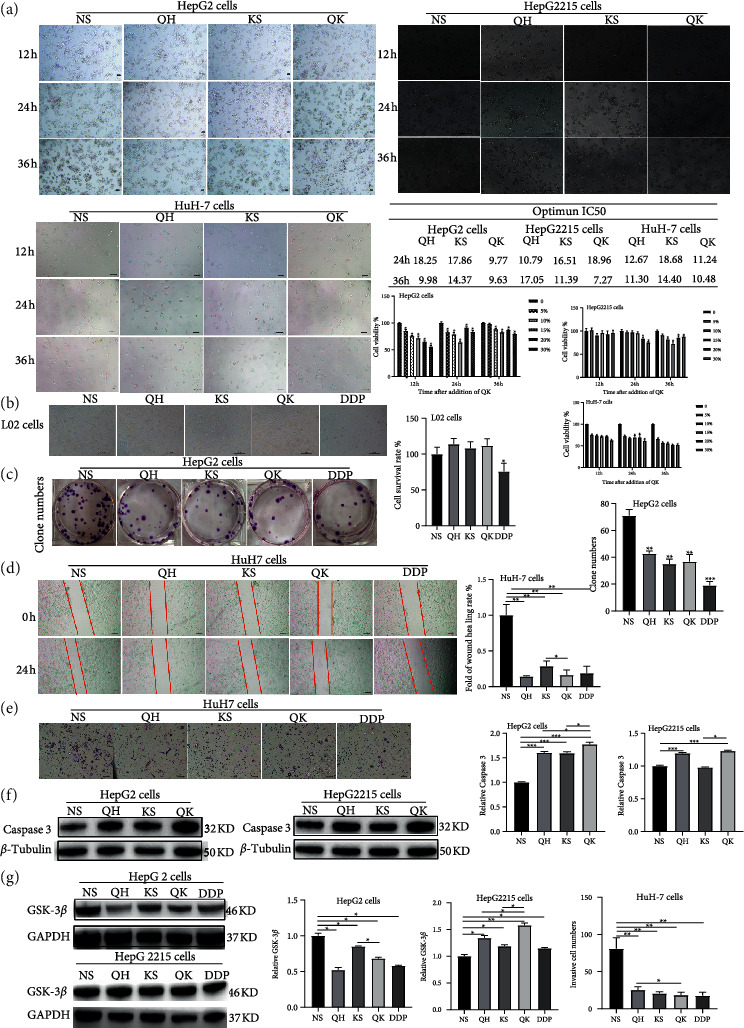
Qinghao-Kushen influenced the level of GSK-3*β* (a) Qinghao-Kushen inhibited the activity of HepG2, HepG2215, and HuH-7 cells. We detected cell numbers at 12-, 24-, and 36-h treatment with different concentrations of drugs, respectively. The image of morphology of the cells was presented after 20% concentrations of drug treatment. The bar chart shows the effect of Qinghao-Kushen on HepG2, HepG2215, and HuH-7 cells' activity. (mean ± SD, *n* = 3). The IC_50_ of drugs was measured at 24 h and 36 h, respectively. (b) Cytotoxic activity of drug-containing serum against L02 cells. The liver L02 cells were treated with different drugs. The drug concentrations were IC_50_ values of HepG2 cells, and the control group was treated with 20% normal saline serum. (c) Qinghao-Kushen inhibited the proliferation of HepG2 cells. HepG2 cells were treated with IC_50_ concentration in Figure 5(a) for 24 h. The control group was treated with 20% normal saline serum. Finally, all experiments were conducted at least three times. A representative result was given. (d) Qinghao-Kushen inhibited the migration capability of HuH-7 cells. HuH-7 cells were treated with different drugs. (e) Qinghao-Kushen inhibited the invasiveness capability of HuH-7 cells. HuH-7 cells were treated with different drugs. (f) The expression of activated caspase 3 was detected with western blot assay. HepG2 and HepG2215 cells were treated with different drugs, and each treatment factor was repeated three times. The *β*-tubulin blot served as the loading control. (g) The expression of GSK-3*β* was detected with western blot assay. HepG2 and HepG2215 cells were treated with different drugs, and each treatment factor was repeated three times. The GAPDH blot served as the loading control. NS, saline medicated serum; QH, Qinghao medicated serum; KS, Kushen medicated serum; QK, Qinghao-Kushen medicated serum; DDP, 5.0 *μ*M cisplatin treatment. ^*∗*^*P* < 0.05, ^*∗*^*P* < 0.01, ^*∗*^*P* < 0.001.

To further verify the molecular mechanism of Qinghao-Kushen, we treated HepG2, HuH-7, and HepG2215 cells with different concentrations of Qinghao-Kushen (0, 5%, 10%, 15%, 20%, and 30%) for 12 h, 24 h, and 36 h. We observed that cell viability decreased gradually with an increased dose of Qinghao-Kushen in HuH-7 and HepG2 cells after 12 h by CCK8 assay (Figure 5(a)). Meanwhile, the inhibitory rate was the highest in HuH-7 and HepG2 cells when the drug concentration was about 12% and 15%, respectively. The 24-h IC_50_ concentration of Qinghao-Kushen was 11.24%, 9.77%, and 18.96% in HuH-7, HepG2, and HepG2215 cells, respectively.

Subsequently, we performed a cytotoxicity experiment to verify the influence of IC_50_ concentration on normal liver L02 cells. The results showed that IC_50_ medicated serum had no inhibitory effect on L02 cells (Figure 5(b)).

Next, to determine the effect of Qinghao-Kushen on long-term colony formation, we treated HepG2 cells with 9.77% Qinghao-Kushen for 24 h and 5.0 *μ*M DDP as a positive control (Figure 5(c)). The result revealed that the number of colonies were lower after the administration of Qinghao-Kushen than the normal saline group. It suggested that Qinghao-Kushen significantly inhibited the growth and proliferation in HepG2 cells.

A wound-healing assay and cell invasion (Figures 5(d) and 5(e)) were used to investigate the effects of Qinghao-Kushen on the motility, migration, and invasiveness in HuH-7 cells. The healing area was relative to 100% in the control group. The Qinghao-Kushen, Qinghao, and Kushen were 16.32% ± 6.07%,14.42% ± 8.2%, and 28.71% ± 7.16%, respectively.

They effectively inhibited the scratch healing area compared with the control group (*P* < 0.01). Moreover, the Qinghao-Kushen (16.32% ± 6.07%) significantly inhibited the scratch healing area than the Kushen group (28.71% ± 7.16%).

The number of cells penetrating out of the compartment membrane in the Qinghao-Kushen, Qinghao group, and Kushen group was 18.50 ± 3.70, 25.50 ± 4.20, and 20.75 ± 2.22, respectively. The number of cells penetrating the compartment membrane was lower than that of the control group (*P* < 0.01). Furthermore, Qinghao-Kushen (18.50 ± 3.70) was similar to the DDP group (17.75 ± 4.57) and significantly inhibited the number of cells infiltrating out of the compartment membrane compared with the Qinghao group (25.50 ± 4.20). These data indicated that Qinghao-Kushen significantly inhibited the migration than Kushen and inhibited cell invasiveness than Qinghao in HuH-7 cells.

The expression level of activated caspase-3 protein was increased and higher after Qinghao-Kushen treatment than Qinghao or Kushen alone in HepG2 and HepG2215 cells (Figure 5(f)). The result showed that Qinghao-Kushen had a synergistic effect on promoting the activated caspase-3 expression in HepG2 and HepG2215 cells. GSK-3*β* was a potential target of Qinghao-Kushen by network analysis. GSK-3*β* expression was decreased after the intervention of Qinghao-Kushen compared with the control group in HepG2 cells but increased GSK-3*β* expression in HepG2215 cells (Figure 5(g)). Collectively, Qinghao-Kushen inhibited cell proliferation and growth in HepG2, HepG2215, and HuH-7 cells. Significantly, Qinghao-Kushen effectively inhibited the migration or invasiveness than Kushen or Qinghao alone in HuH-7 cells. Meanwhile, the synergistic effect of Qinghao-Kushen increased GSK-3*β* expression more than alone in HepG2215 cells.

### 3.7. The Effects of Matrine-Scopoletin, Active Ingredients, in Qinghao-Kushen on the GSK-3*β* Expression in Liver Cancer Cells

**Figure 6:**
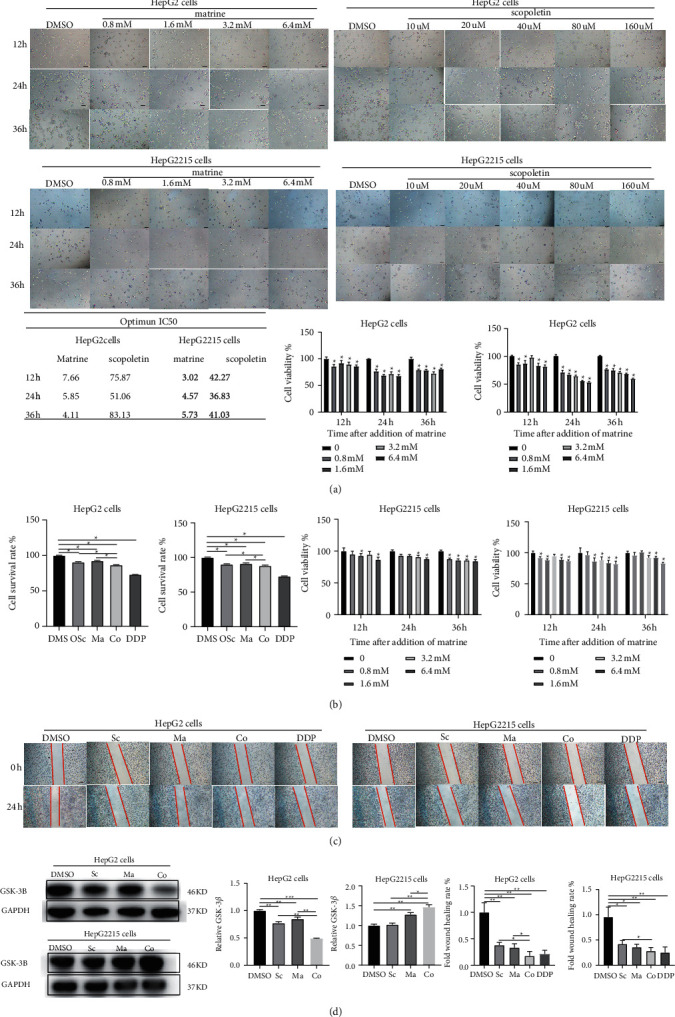
Matrine and scopoletin influenced the level of GSK-3*β*. (a) Matrine and scopoletin inhibited the activity of HepG2 and HepG2215 cells. We detected cell numbers at 12-, 24-, and 36-h treatment with different concentrations of drugs, respectively. The bar chart shows the effect of matrine and scopoletin on HepG2 and HepG2215 cell activity. (mean ± SD, *n* = 3). The IC_50_ of drugs was measured at 12 h, 24 h, and 36 h, respectively. (b) Matrine-scopoletin inhibited the activity of HepG2 and HepG2215 cells. (c) Matrine and scopoletin inhibited the migration capability of HepG2 and HepG2215 cells. Cells were treated with different drugs. (d) The expression of GSK-3*β* was detected with western blot assay. HepG2 and HepG2215 cells were treated with different drugs, and each treatment factor was repeated three times. The GAPDH blot served as the loading control. DMSO, dimethyl sulfoxide; Ma, matrine; Sc, scopoletin; Co, matrine-scopoletin; DDP, 5.0 *μ*M cisplatin treatment. ^*∗*^*P* < 0.05, ^*∗*^*P* < 0.01, ^*∗*^*P* < 0.001.

To test whether matrine and scopoletin, active ingredients, in Qinghao-Kushen regulated GSK-3*β* expression, HepG2 and HepG2215 cells were treated with various concentrations of matrine (0 mM, 0.8 mM, 1.6 mM, 3.2 mM and 6.4 mM) and scopoletin (0 *μ*M, 10 *μ*M, 20 *μ*M, 40 *μ*M, 80 *μ*M and 160 *μ*M).The 24-h IC_50_ of matrine and scopoletin was about 5.85 mM and 51.06 *μ*M in HepG2, while 4.57 mM and 36.83 *μ*M in HepG2215 cells (Figure 6(a)). To further confirm the effect of matrine-scopoletin on liver cancer cells, we used DPS software for uniform design, then combined it with the CCK8 method to calculate the 24-h IC_50_ of the matrine-scopoletin interventions in HepG2 and HepG2215 cells. The best ratio of matrine and scopoletin was 1.6 mM: 40 *μ*M in HepG2 cells, while was 4 mM: 40 *μ*M in HepG2215 cells. CCK8 results showed that matrine, scopoletin, and matrine-scopoletin inhibited cell viability in HepG2 and HepG2215 cells. Critically, the combination of matrine-scopoletin was more effective than alone (Figure 6(b)).

Subsequently, we observed that matrine, scopoletin, and matrine-scopoletin reduced scratch healing area more than the DMSO group. Importantly, the inhibition rate of cell healing in the matrine-scopoletin group was more pronounced than in matrine or scopoletin in HepG2 or HepG2215 cells. The intervention effect was similar to DDP (Figure 6(c)). Notably, matrine-scopoletin inhibited the proliferation and migration better than matrine or scopoletin alone in HepG2 and HepG2215 cells. A similar result was shown on the expression of GSK-3*β* protein. GSK-3*β* protein was decreased after the matrine-scopoletin intervention in HepG2 cells (Figure 6(d)). In contrast, matrine-scopoletin increased GSK-3*β* expression in HepG2215 cells than matrine and scopoletin alone. Together, matrine and scopoletin inhibited cell proliferation and growth in HepG2 and HepG2215 cells. Meanwhile, marine, scopoletin, and marine-scopoletin degraded the expression of GSK-3*β* in HepG2 cells while elevated GSK-3*β* in HepG2215 cells.

## 4. Discussions

This study confirmed that Qinghao, Kushen, and Qinghao-Kushen inhibited the proliferation and viability in HuH-7, HepG2, and HepG2215 cells. A hallmark of HBV infection is the production of stable and long-lasting cccDNA viral in the nuclei of infected hepatocytes. No drug effectively eliminated HBV cccDNA [33, 34], which is considered a significant barrier to treating chronic HBV infection and reducing the risk of hepatocellular carcinoma. So, we selected HepG2215 cells for the study to find more effective drugs for the treatment of liver cancer with HBV infection. To our knowledge, we formed a drug pair of Qinghao-Kushen for the first time. The mechanism of Qinghao-Kushen was also analyzed by network pharmacology in HCC. Qinghao or Kushen, as a kind of classical, traditional Chinese medicine, has been widely studied previously in the antitumor field. Still, the effect of the Qinghao-Kushen combination on HCC remains unclear. We firstly found that Qinghao-Kushen significantly inhibited migration more than Kushen alone and inhibited invasiveness than Qinghao alone in HuH-7 cells. Furthermore, we discovered that matrine-scopoletin, two active ingredients by LC-MS in Qinghao-Kushen, synergistically inhibited the viability in HepG2 and HepG2215 cells, which was more effective than alone.

Qinghao-Kushen increased activated caspase-3 expression in HepG2 and HepG2215 cells. Furthermore, the increase was more evident than Qinghao or Kushen in HepG2 cells. Activated caspase-3 as a marker of apoptosis indicate that Qinghao-Kushen can induce apoptosis. We validated matrine and scopoletin targeting GSK-3*β*, a key molecule in the treatment of liver cancer. The combination of the matrine-scopoletin elevated the expression of GSK-3*β* in HepG2215 cells compared with matrine or scopoletin alone. Scopoletin, a coumarin compound isolated from *Artemisia* species and other plant genera [35], is another bioactive compound in *Artemisia annua* [36]. Studies showed that scopoletin had antitumor activity and inhibited the migration of HeLa cells [37]. Matrine was a natural alkaloid extracted from *Sophora flavescens* [38]. It reported [39] that matrine inhibited the clone formation and proliferation in HepG2 cells, which may be related to the inhibition of the Akt/GSK3*β*/*β*-catenin signaling pathway. Therefore, the main compounds of Qinghao-Kushen in the treatment of HCC may be related to matrine and scopoletin. Interestingly, Qinghao-Kushen produced new compounds during metabolisms, such as O-methoxycatechol-O-sulfate and 2-Methoxyacetaminophen sulfate, which belong to organic sulfuric acids and derivatives. GSK-3*β*, AKT1, STAT3, mTOR, and EGFR were core targets in the H-C-T-P network. In summary, these results indicated that Qinghao-Kushen was associated with the treatment for HCC through multiple targets. In this article, we only verified GSK-3*β* because of its core position in the network. Furthermore, five compounds in Qinghao-Kushen targeted GSK-3*β*, which was involved in the process of multiple cancer pathways. The roles of GSK-3*β* in HCC remain controversial. Some studies showed that GSK-3*β* acted as a tumor suppressor gene in HCC [40] and negatively regulated Wnt/*β*-catenin. The inhibition of GSK-3*β* leads to nuclear accumulation of *β*-catenin [25] and contributes to hepatic transformation [24]. A study demonstrated the GSK-3*β* inactivation by HBx upregulated cyclin D1. Cyclin D1 further causes HCC progression [41]. HBx upregulated cyclin D1 in HepG2215 cells [42]. In addition, it showed that HBx increased cyclin D1 through ERK-mediated inactivation of GSK-3*β* [43]. Given the important role of GSK-3*β* in cyclin D1 degradation [44], GSK-3*β* has a tumor suppressor function in HepG2215 cells. The above results indicated that HBx may affect the activity of GSK-3*β* in HepG2215 cells, leading to the progression of HCC. Qinghao-Kushen and matrine-scopoletin, the active ingredient of Qinghao-Kushen, enhanced GSK-3*β* expression, a beneficial gene for treating HBV-related HCC. However, the GSK-3*β* expression in HepG2 differed from that in HepG2215 cells. Some reports have shown high expression of GSK-3*β* in many tumors, including liver [45], colon [46], and ovarian pancreatic tumors [47]. A study showed that the expression of GSK-3*β* in the HepG2 cell line was increased [48]. After using different concentrations of GSK-3*β* inhibitor, the viability and proliferation ability in HepG2 cells decreased compared with the control group [48]. These data suggested that decreased GSK-3*β* activity significantly reduced the viability and proliferative capacity in HepG2 cells. Consistently, our study showed that Qinghao, Kushen, Qinghao-Kushen, matrine, scopoletin, and matrine-scopoletin reduced GSK-3*β* expression in HepG2 cells. The inhibition of GSK-3*β* activity inhibited liver cancer progression, and this anticancer effect was mainly achieved through the AMPK/mTOR signaling pathway [49]. Moreover, hyperactive mTOR promoted the advancement of HCC through augmented GSK-3*β*/MMPs [50]. In our study, mTOR was one of the targets of Qinghao-Kushen. The relationship between GSK-3*β* and mTOR required further investigations. Nevertheless, further studies are required to examine the expression of GSK-3*β* in other HCC cells or HCC tissues. Briefly, consistent with the results of network pharmacology analysis, Qinghao-Kushen and its active compounds scopoletin and matrine all targeted GSK-3*β*.

## 5. Conclusion

In this study, we formed Qinghao-Kushen herb pairs and preliminarily analyzed the possibility of Qinghao-Kushen creating a drug pair. Matrine and scopoletin were pivotal compounds in Qinghao-Kushen. We validated that Qinghao-Kushen decreased the proliferation and viability ability of HuH-7, HepG2, and HepG2215 cells in vitro. Moreover, the effect of the Qinghao-Kushen combination was significantly better than that of single Qinghao or single Kushen in HepG2 and HuH-7 cells. Qinghao, Kushen, matrine, and scopoletin degraded GSK-3*β* in HepG2 cells, whereas they upregulated GSK-3*β* expression in HepG2215 cells, which may be a potential target of HCC.

## Figures and Tables

**Table 1:** Network meta-analysis of the overall efficacy of the 12 Chinese medicine on patients with HCC (OR 95%CI). The column intervention is compared with the row intervention (lower left portion). An OR of more than 1 favors column -defining treatment for the clinical total effective rate. Numbers on red are statistically significant. The ORs for comparisons in the opposite direction was also conducted (upper right portion). (e.g., the OR for Shenpei compared with Xiaoyao is 1/1.49 = 0.67).

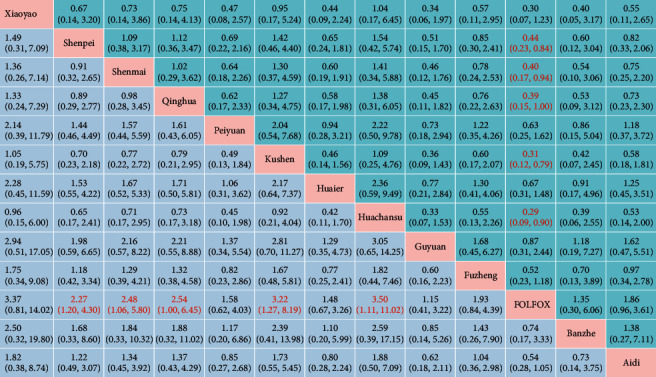

## Data Availability

The data used to support the findings of this study are included within the article.

## Abbreviations

HCC: Hepatocellular carcinoma
TCM: Traditional Chinese medicine
DHA: Dihydroartemisinin
GSK-3*β*: Glycogen synthase kinase 3 beta
LC-MS: Liquid chromatography mass spectrometry
FOLFOX: Oxaliplatin-based chemotherapy drugs
DDP: Cisplatin
mTOR: Mammalian target of rapamycin.

## Supplementary Materials

Supplementary Materials Figure S1: Total ion chromatogram of the sample. (A) Total ESI(+) ion diagram of the quality control sample. (B) Total ESI(−) ion diagram of the quality control sample. M1: Qinghao medicated serum. M2: Kushen medicated serum. M3: Qinghao- Kushen medicated serum. M4: Normal saline serum. Table S1: Systematic search and screening process of trials. Table S2: Studies included in the multiple treatment meta-analysis. Table S3: The SUCRA results of different treatment relative ranking. Table S4: Metabolized compounds. The [DATA TYPE] data used to support the findings of this study are included within the article.
