# Supplementary material for: The Effects of Qinghao-Kushen and Its Active Compounds on the Biological Characteristics of Liver Cancer Cells
Source: Evid Based Complement Alternat Med. 2022 Jun 10;2022:8763510. doi: 10.1155/2022/8763510 (PMC9205744; doi:10.1155/2022/8763510)
Supplement: Supplementary Materials — Figure S1: Total ion chromatogram of the sample. (A) Total ESI(+) ion diagram of the quality control sample. (B) Total ESI(−) ion diagram of the quality control sample. M1: Qinghao medicated serum. M2: Kushen medicated serum. M3: Qinghao- Kushen medicated serum. M4: Normal saline serum. Table S1: Systematic search and screening process of trials. Table S2: Studies included in the multiple treatment meta-analysis. Table S3: The SUCRA results of different treatment relative ranking. Table S4: Metabolized compounds. The [DATA TYPE] data used to support the findings of this study are included within the article. [file 8763510.f1.zip › 8763510.f1/Table S2 (1).pdf]

Table S 2 Studies included in the multiple-treatments meta-analysis							
	Number of cases		Overall efficiency		Interventions		
Author year	Experimentalgroup	Control group	Experimentalgroup	Control group	Experimental group	Control group	Outcome indicator
Tang 2016	54	54	22	13	Sheimai+FOLFOX	FOLFOX	①②③④
He 2018	37	37	19	13	Aidi+FOLFOX	FOLFOX	③
Cheng 2012	36	36	18	13	Aidi+FOLFOX	FOLFOX	②③④
Chen 2013	38	40	31	26	Shenpei+FOLFOX	FOLFOX	③④
Hu 2016	40	40	24	18	Sheipei+FOLFOX	FOLFOX	①②③
Tian 2015	30	30	16	4	Huachansu+FOLFOX	FOLFOX	②③④
Li 2013	38	38	16	12	Peiyuan+FOLFOX	FOLFOX	②③④
Ding 2015	38	37	22	13	Qinghua+FOLFOX	FOLFOX	③④
Yuan 2012	19	19	9	4	Xiaoyao+FOLFOX	FOLFOX	②③④
Wang 2012	24	31	4	4	Banzhe+FOLFOX	FOLFOX	③④
Xia 2017	28	28	12	11	Huaier+ FOLFOX	FOLFOX	②③
Zhao 2018	60	38	35	16	Fuzheng+FOLFOX	FOLFOX	③④
Yang 2015	40	40	10	9	Guyuan+FOLFOX	FOLFOX	③④
Huang 2018	40	40	29	18	Kushen+FOLFOX	FOLFOX	③⑤
Zhu 2017	30	30	10	6	Huaier+FOLFOX	FOLFOX	②③⑤

①Cellular immune index②KPS score③Overall efficiency④Toxic and side effect⑤liver function
