# Supplementary material for: The Effects of Qinghao-Kushen and Its Active Compounds on the Biological Characteristics of Liver Cancer Cells
Source: Evid Based Complement Alternat Med. 2022 Jun 10;2022:8763510. doi: 10.1155/2022/8763510 (PMC9205744; doi:10.1155/2022/8763510)
Supplement: Supplementary Materials — Figure S1: Total ion chromatogram of the sample. (A) Total ESI(+) ion diagram of the quality control sample. (B) Total ESI(−) ion diagram of the quality control sample. M1: Qinghao medicated serum. M2: Kushen medicated serum. M3: Qinghao- Kushen medicated serum. M4: Normal saline serum. Table S1: Systematic search and screening process of trials. Table S2: Studies included in the multiple treatment meta-analysis. Table S3: The SUCRA results of different treatment relative ranking. Table S4: Metabolized compounds. The [DATA TYPE] data used to support the findings of this study are included within the article. [file 8763510.f1.zip › 8763510.f1/Table S3 (1).pdf]

Table S 3 The SUCRA results of different treatment relative ranking

Treatment	SUCRA	PrBest	MeanRank
FOLFOX	12.6	0	12.4
Aidi	46.8	0.4	7.9
Banzhe	34.4	2.7	9.5
Fuzheng	49	1.8	7.6
Guyuan	25.3	0.3	10.7
Huachansu	77.5	25.7	3.9
Huaier	38.9	3.9	8.9
Kushen	77	19	4
Peiyuan	40.4	1.2	8.7
Qinghua	64.2	7.8	5.7
Shenmai	64.1	5.9	5.7
Shenpei	59.5	2.3	6.3
Xiaoyao	71.4	25.3	4.7
