# Supplementary material for: The Effects of Qinghao-Kushen and Its Active Compounds on the Biological Characteristics of Liver Cancer Cells
Source: Evid Based Complement Alternat Med. 2022 Jun 10;2022:8763510. doi: 10.1155/2022/8763510 (PMC9205744; doi:10.1155/2022/8763510)
Supplement: Supplementary Materials — Figure S1: Total ion chromatogram of the sample. (A) Total ESI(+) ion diagram of the quality control sample. (B) Total ESI(−) ion diagram of the quality control sample. M1: Qinghao medicated serum. M2: Kushen medicated serum. M3: Qinghao- Kushen medicated serum. M4: Normal saline serum. Table S1: Systematic search and screening process of trials. Table S2: Studies included in the multiple treatment meta-analysis. Table S3: The SUCRA results of different treatment relative ranking. Table S4: Metabolized compounds. The [DATA TYPE] data used to support the findings of this study are included within the article. [file 8763510.f1.zip › 8763510.f1/Table S4 (1).pdf]

31 metabolize compounds in Qinghao-Kushen.a shows active compounds of Qing hao derived from SymMap, b shows active compounds of Kushen derived from SymMap.

Table S 4 metabolize compounds

Sample	Metabolite	m/z	Adducts	Formula	Fragmentation Score	Theoretical Fragmentation Score	Mass Error (ppm)	Structure
Qing hao, Qing hao-Kushen	2-Hydroxy-4-(4-methoxyphenyl)-1H-phenalen-1-one	301.0891263	M-H, M+Na-2H	C20H14O3	-	55.6	6.979414012	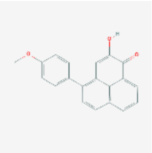
Qing hao, Qing hao-Kushen	Coumaroyl Hexoside	325.0913887	M-H	C15H18O8	38.3	-	-4.60711614	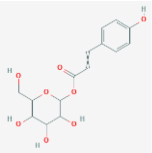
Qing hao, Qing hao-Kushen	(R)-Pantothenic acid 4'-O-b-D-glucoside	402.1396885	M+Na-2H	C15H27NO10	-	31.6	3.999769063	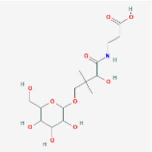
Qing hao, Qing hao-Kushen	{[(2Z)-2-(phenylmethylidene)heptyl]oxy}sulfonic acid	329.1048658	M+FA-H	C14H20O4S	-	62.9	-5.51607972	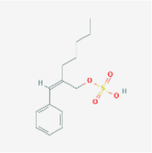
Qing hao, Qing hao-Kushen	Byssochlamic acid	313.1100841	M-H2O-H	C18H20O6	-	33.8	5.831561207	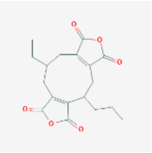
Qing hao,Qing hao-Kushen	3,4,5-trihydroxy-6-(2,4,6-trihydroxyphenoxy)oxane-2-carboxylic acid	317.0539687	M-H	C12H14O10	-	58.6	8.012585458	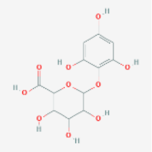
Qing hao,Qing hao-Kushen	9-Oxo-nonanoic acid	217.1068954	M+FA-H	C9H16O3	-	76.8	-7.27367658	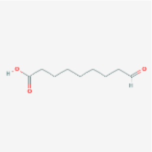

Qing hao,Qing hao-Kushen	Furaneol 4-(6-malonylglucoside)	357.0811553	M-H2O-H	C15H20O11	-	82.0	-4.16116267	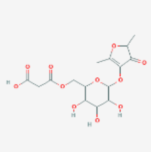
Qing hao,Qing hao-Kushen	Questiomycin A	249.0070277	M+K-2H	C12H8N2O2	-	57.0	-0.73109972	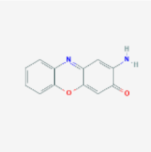
Qing hao,Qing hao-Kushen	3,7,8,15-Scirpenetetrol	299.1483039	M+H-H2O, M+H, M+Na, M+H-2H2O	C15H22O6	-	63.3	-2.14289928	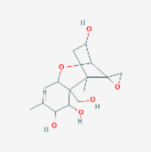
Qing hao,Qing hao-Kushen	2-Pentanamido-3-phenylpropanoic acid	250.1432414	M+H-H2O, M+H	C14H19NO3	-	63.1	-1.32099671	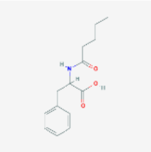
Qing hao,Qing hao-Kushen	Scopoletin [a]	193.049228	M+H	C10H8O4	89.9	-	-1.59952902	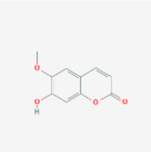
Qing hao,Qing hao-Kushen	3-[4-hydroxy-3-(3-methylbut-2-en-1-yl)phenyl]prop-2-enoic acid	250.1432395	M+NH4	C14H16O3	-	75.3	-2.2853312	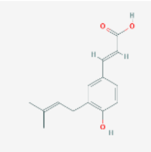
Qing hao,Qing hao-Kushen	Thalictroidine	234.1484272	M+H	C14H19NO2	59	-	-1.83631858	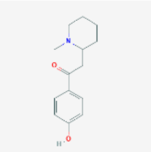
Qing hao,Qing hao-Kushen	Carnosine	271.0781986	M+2Na-H	C9H14N4O3	33.6	-	1.960885798	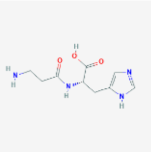

Ku shen,Qing hao-Kushen	Piscidic acid	255.0498553	M-H	$C_{11}H_{12}O_7$	86.7	-	-4.57319829	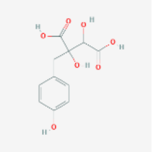
Ku shen,Qing hao-Kushen	(-)-Epigallocatechin sulfate	367.0112171	M-H2O-H	$C_{15}H_{14}O_{10}S$	-	75.7	-4.4281108	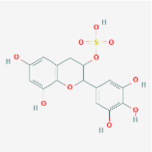
Ku shen,Qing hao-Kushen	5-benzyloxolan-2-one	213.0311155	M+K-2H	$C_{11}H_{12}O_2$	-	73.3	-6.92490863	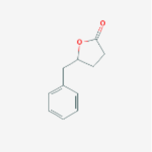
Ku shen,Qing hao-Kushen	Ginkgolide J	445.1120202	M+Na-2H	$C_{20}H_{24}O_{10}$	-	71.4	0.95590232	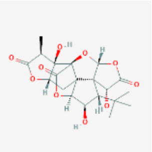
Ku shen,Qing hao-Kushen	{3-[3-(3,4-dihydroxy-2-methoxyphenyl)prop-2-enoyl]phenyl}oxidanesulfonic acid	365.0319474	M-H	$C_{16}H_{14}O_8S$	-	61.5	-4.68421072	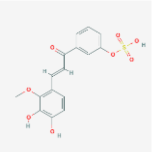
Ku shen,Qing hao-Kushen	Matrine [b]	249.1954437	M+H, 2M+H	$C_{15}H_{24}N_2O$	68.2	-	-2.80467685	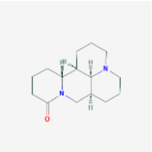
Ku shen,Qing hao-Kushen	Bufotenin	205.1332053	M+H	$C_{12}H_{16}N_2O$	-	43.9	-1.63776558	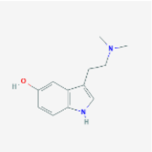
Ku shen,Qing hao-Kushen	N-(4-aminobutyl)-3-(4-hydroxyphenyl)propanimidic acid	259.143614	M+Na	$C_{13}H_{20}N_2O_2$	-	61.7	8.11093642	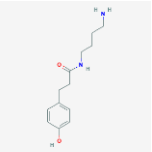

Ku shen,Qing hao-Kushen	Argyrolobine	263.1748236	M+H	C15H22N2O2	64.5	-	-2.21530158	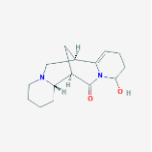
Ku shen,Qing hao-Kushen	1-(9H-Pyrido[3,4-b]indol-1-yl)-1,4-butanediol	257.1278155	M+H	C15H16N2O2	39.5	-	-2.49381854	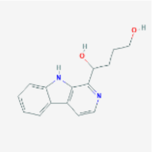
Ku shen,Qing hao-Kushen	Tryptophan betaine	288.1699072	M+ACN+H	C14H18N2O2	48	-	-3.03144446	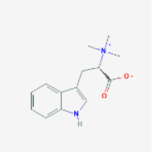
Ku shen,Qing hao-Kushen	Piperonal	151.0386834	M+H	C8H6O3	34.8	-	-1.91344056	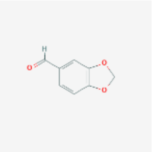
Ku shen,Qing hao-Kushen	(+)-Setoclavine	272.1750885	M+NH4	C16H18N2O	-	46.7	-2.55874874	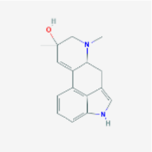
Ku shen,Qing hao-Kushen	13-Hydroxylupanine	265.1905315	M+H	C15H24N2O2	65.2	-	-1.9795088	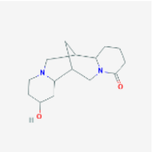
Qing hao-Kushen	O-methoxycatechol-O-sulphate	203.0005959	M-H	C7H8O5S	-	39.4	-6.7251021	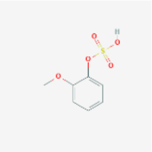
Qing hao-Kushen	2-Methoxyacetaminophen sulfate	242.0116758	M-H2O-H	C9H11NO6S	-	80.1	-4.56340963	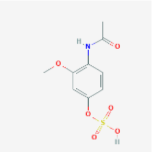
