# Supplementary figures and images for: The Effects of Qinghao-Kushen and Its Active Compounds on the Biological Characteristics of Liver Cancer Cells
Source: Evid Based Complement Alternat Med. 2022 Jun 10;2022:8763510. doi: 10.1155/2022/8763510 (PMC9205744; doi:10.1155/2022/8763510)

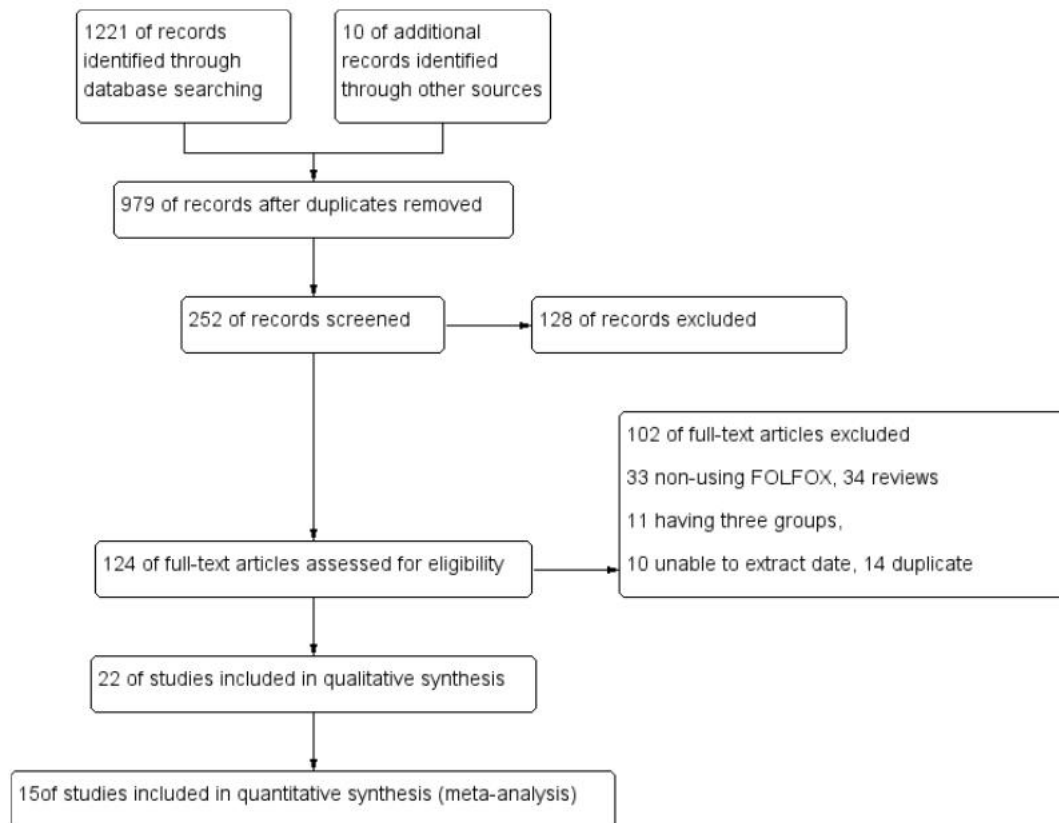

Table S 1 Systematic search and screening process of trials.

Supplement: Supplementary Materials — Figure S1: Total ion chromatogram of the sample. (A) Total ESI(+) ion diagram of the quality control sample. (B) Total ESI(−) ion diagram of the quality control sample. M1: Qinghao medicated serum. M2: Kushen medicated serum. M3: Qinghao- Kushen medicated serum. M4: Normal saline serum. Table S1: Systematic search and screening process of trials. Table S2: Studies included in the multiple treatment meta-analysis. Table S3: The SUCRA results of different treatment relative ranking. Table S4: Metabolized compounds. The [DATA TYPE] data used to support the findings of this study are included within the article. [file 8763510.f1.zip › 8763510.f1/Table S1 (1).pdf]
